# The Effects of Different Training Backgrounds on VO_2_ Responses to All-Out and Supramaximal Constant-Velocity Running Bouts

**DOI:** 10.1371/journal.pone.0133785

**Published:** 2015-08-07

**Authors:** Rafael Alves de Aguiar, Felipe Domingos Lisbôa, Tiago Turnes, Rogério Santos de Oliveira Cruz, Fabrizio Caputo

**Affiliations:** Human Performance Research Group, Center for Health and Sport Science, Santa Catarina State University, Florianópolis, Santa Catarina State, Brazil; Norwegian University of Science and Technology, NORWAY

## Abstract

To investigate the impact of different training backgrounds on pulmonary oxygen uptake (V̇O_2_) responses during all-out and supramaximal constant-velocity running exercises, nine sprinters (SPRs) and eight endurance runners (ENDs) performed an incremental test for maximal aerobic velocity (MAV) assessment and two supramaximal running exercises (1-min all-out test and constant-velocity exercise). The V̇O_2_ responses were continuously determined during the tests (K4^b2^, Cosmed, Italy). A mono-exponential function was used to describe the V̇O_2_ onset kinetics during constant-velocity test at 110%MAV, while during 1-min all-out test the peak of V̇O_2_ (V̇O_2_peak), the time to achieve the V̇O_2_peak (tV̇O_2_peak) and the V̇O_2_ decrease at last of the test was determined to characterize the V̇O_2_ response. During constant-velocity exercise, ENDs had a faster V̇O_2_ kinetics than SPRs (12.7 ± 3.0 vs. 19.3 ± 5.6 s; *p* < 0.001). During the 1-min all-out test, ENDs presented slower tV̇O_2_peak than SPRs (40.6 ± 6.8 and 28.8 ± 6.4 s, respectively; *p* = 0.002) and had a similar V̇O_2_peak relative to the V̇O_2_max (88 ± 8 and 83 ± 6%, respectively; *p* = 0.157). Finally, SPRs was the only group that presented a V̇O_2_ decrease in the last half of the test (-1.8 ± 2.3 and 3.5 ± 2.3 ml.kg^-1^.min^-1^, respectively; *p* < 0.001). In summary, SPRs have a faster V̇O_2_ response when maximum intensity is required and a high maximum intensity during all-out running exercise seems to lead to a higher decrease in V̇O_2_ in the last part of the exercise.

## Introduction

Short and intense running exercise requires substantial contributions from both aerobic and anaerobic systems [1]. But, a high accumulation of metabolites derived from anaerobic metabolism can impair running performance [2]. Therefore, modifications in the oxygen uptake (V̇O_2_) response, affecting energy metabolism and the extent to which the intra-muscular milieu is perturbed, are useful for improving performance in this kind of exercise [3].

During moderate and heavy constant work-rate exercise, endurance athletes (ENDs) have faster pulmonary V̇O_2_ kinetics than sprinters (SPRs) [4–6], which increases the aerobic contribution from the onset of exercise. However, during competitive running events, speed varies throughout a race (specifically with higher start speeds) [7–9]; therefore, constant work-rate exercises do not represent a “real life” performance. Surprisingly, to our knowledge, no previous study has compared V̇O_2_ responses between ENDs and SPRs during long sprint running performances, though it seems to be one of the factors setting maximal performances in track running [10]. The short nature of performances around 1-min requires the subjects to maintain nearly maximum effort throughout the exercise [11], demanding that their anaerobic systems approach maximal rates of ATP resynthesis in the active muscles. This can lead to a potential speed-up in the V̇O_2_ response [9, 12] since for some models the changes per unit time in V̇O_2_ is proportional to the rate of phosphocreatine (PCr) breakdown in the active muscles per unit of change in time (i.e., the *∆[PCr]/∆t* ratio) [13, 14]. While ENDs have shown faster V̇O_2_ kinetics for constant work-rate exercises [6, 15], SPRs might be more prone to speed-up the V̇O_2_ kinetics during all-out exercises than ENDs due to faster PCr breakdown [16, 17].

On the other hand, the higher anaerobic ATP turnover at the onset of all-out exercise in SPRs may also cause different impacts on the subsequent V̇O_2_ compared to the ENDs. Recent studies have shown that a fast start strategy leads to a decrease in V̇O_2_ toward the end of races [8, 9, 18] as a result of metabolic perturbations related to acidosis [8]. Thus, the larger anaerobic contribution of SPRs during early spurt of the all-out test [19, 20] induces a higher accumulation of metabolites [21] leading to a higher decrease in the V̇O_2_ during the last part of the exercise.

The purpose of this study was to investigate the impact of different training backgrounds (i.e., SPRs vs. ENDs) and metabolic perturbations on the V̇O_2_ responses during all-out and supramaximal constant-velocity running exercises. We hypothesized that the SPRs would have faster V̇O_2_ responses compared to the ENDs during all-out exercise. Conversely, the ENDs would have faster V̇O_2_ response during the constant-velocity exercise and a lower V̇O_2_ decrease during the last part of the all-out exercise compared to the SPRs. This cross-sectional study was a feasible way to assess the manner and degree to which each training background and the different pacing strategies affect V̇O_2_ responses, providing further information for the determinants of V̇O_2_ kinetics during supramaximal exercise.

## Materials and Methods

### Subjects

Seventeen men volunteered to participate in this study and provided written informed consent after a thorough explanation of the study protocol. This investigation was approved by the Santa Catarina State University Research Ethics Committee. This work was performed according to the Declaration of Helsinki. Subjects with different training status were deliberately targeted for this study, and were assigned to two groups. The first group was composed of eight endurance athletes (28.6 ± 4.2 yr, 176 ± 6 cm, 70.7 ± 8.1 kg), and the second group was composed of nine sprinters (19.6 ± 3.9 yr, 180 ± 7 cm, 78.5 ± 8.7 kg). The ENDs comprised 3 tri-athletes and 5 distance runners. The distance runners were specialists in 3000 m steeple chase events (1), 5–10 km (2) or marathon (2). The SPRs comprised 1 decathlete, 1 long jumper, 1 high jumper and 6 track sprinters. The track sprinters were specialists in 100 (4) or 400 m (2). All ENDs and SPRs participate in 10 km and 100 m events, respectively, during the year. The athletes competed at regional and/or national level events and trained for at least 1.5 years about 10 hours per week. Seasonal best performance time for SPRs on 100 m was 11.07 (range, 10.51–11.67 s) and for ENDs on 10 km was 35.34 min (range, 31.00–38.10 min). The personal best time corresponded to 86.7 ± 3.0 and 74.4 ± 5.8% of the current 100 m and 10 km world record, respectively.

### Procedures

Three testing sessions were performed on a synthetic running track over a 2-week period. The first test was an incremental test to determine V̇O_2_max and maximal aerobic velocity (MAV). The following tests were a 1-min all-out test (1MT) and a constant-velocity running bout at 110%MAV, which were performed randomly. Prior to the 1MT and 110%MAV, subjects performed a pre-test warm-up consisting of 5-min running at approximately 65%MAV followed by three practice sprints of 5-10-m (interspersed with 20-s of jogging), and then, rested for 5-min. All sessions were performed at the same time of day (±2 h) to minimize the effects of diurnal variations on the measured variables. Subjects trained during the experimental period, but were directed to arrive fully rested for the experimental sessions. Moreover, the subjects were asked to abstain from products that contained caffeine or alcohol on the test day.

### Materials

During each test the respiratory gas exchange variables were collected, using a breath-by-breath portable gas analyzer (Cosmed K4^b2^, Rome, Italy). The portable unit and the battery were fixed to the participant by a body harness. Calibration procedures were performed before each test, according to the manufacturer’s recommendations.

Capillary blood samples were taken for the determination of amount of blood lactate accumulated (∆BLC) in 1MT and 110%MAV. ∆BLC was calculated subtracting BLC immediately prior to the test from maximal BLC. While blood samples during incremental test and 110%MAV were collected at rest, immediately, 3, 5 and 7-min post tests, during 1MT blood samples were collected at rest, immediately prior to the test and for 60-min post-test (every 1-min from 0 to 10, every 2-min from 10 to 20 and every 5-min from 20 to 60 min). Arterialized capillary blood (25 μL) was sampled by micropuncture at the earlobe, and then stored at eppendorf tubes containing 50 μL of 1% NaF in a -30°C environment. Later, samples were analyzed by enzyme electrode technology (YSI 1500 SPORT, Yellow Springs, Ohio, USA). Before and after (3, 5 and 7-min) 1MT and 110%MAV, blood samples also were taken from a finger in heparinized capillary tube (150 μL) to analyze blood pH. These blood samples were immediately capped, gently agitated, and then stored in an ice chest. Within 15-min of collection, all blood samples were analyzed at 37°C (GEM Premier 3000, Instrumentation Laboratory, Lexington, USA). Decrease in blood pH (∆pH) was calculated as pH before the test minus the lowest pH after test.

### Incremental test and supramaximal constant-velocity exercise

The Incremental test began at a velocity of 8.5 km.h^-1^ and was increased by 0.5 km.h^-1^ per minute until the participant terminated the test owing to volitional exhaustion. The subjects adjusted their running velocity to auditory signals at 20-m intervals, delimited by cones along the track. All subjects were encouraged to put forth their best effort. To determine the V̇O_2_max, the V̇O_2_ was reduced to 15-s average values during the incremental test, and the highest 15-s V̇O_2_ value reached was considered the subject’s V̇O_2_max. MAV was calculated as the velocity of the last stage fully completed, plus, if necessary, the fraction of time spent multiplied by 0.5 km.h^-1^ in the stage at which exhaustion occurred.

The supramaximal constant-velocity exercise bout was performed twice at 110%MAV in the same day within a minimum interval of 1 h. During the tests, the subjects maintained a constant-velocity according to the same auditory pacing procedure as described previously. The subjects performed the tests until volitional exhaustion or if they were stopped because they were more than 2-m late compared to the expected pace.

### 1-min all-out test

During 1MT the subjects were instructed to maintain their velocity as high as possible throughout the entire test. Verbal encouragement was provided as a motivation; however, the subjects were neither informed of the elapsed time nor of the remaining time to discourage pacing. The subjects ran on the inside lane, and performance variables were recorded by a digital video at a rate of 30 Hz (SONY DCR-SR68; Sony, Tokyo, Japan). The maximal velocity (V_max_) was analysed by a camera, which was placed perpendicular to the direction of the cones placed every 5 m within the 20–60-m track, and was considered the highest mean velocity recorded between the cones. The mean velocity (V_mean_) during the 1MT was calculated as the total distance covered (m) divided by 60s. The total distance covered was analysed using orange cones that were placed every 20 m along the track, and it was considered as the amount of cones overcome multiplied by 20 plus the distance exceeding the last cone, if necessary.

### V̇O_2_ analysis

The breath-by-breath V̇O_2_ data from each test were initially examined to exclude occasional errant breath values, i.e. values lying more than three standard deviations outside the local mean (i.e. five-point rolling mean). To measure the V̇O_2_rest, the participants remained standing for 5-min prior to the test, and the V̇O_2_ of the last 2 min were averaged.

To characterize the overall V̇O_2_ kinetics during the 110%MAV, data were interpolated to give one value per second and time aligned. Data were then averaged across the two tests from 120 s prior to the onset of exercise to the end-point of the shorter of the two tests. Finally, a mono-exponential function was used to describe the V̇O_2_ response during 110%MAV:

$$V{\text{O}}_{2}\;\left(\text{t}\right)\;=\;{\text{VO}}_{2}rest+\;\text{A}(1\;-\;{\text{e}}^{-\left(\frac{t-TD}{tau}\right)})$$ (1)

where V̇O_2_(t) is V̇O_2_ at time t, V̇O_2_rest is the pre-test V̇O_2_; A is the amplitude of the increase in V̇O_2_ above the pre-test value; TD is the delay between the start of the square wave and the onset of primary component, and tau is the time constant of the exponential response that comprises the primary component. The cardiodynamic phase or phase I was excluded from the analysis based on previous visual inspection. This visual inspection of the individual V̇O_2_ responses revealed that the first phase typically lasted less than 15 s. Hence we excluded just the first 15 s of data from the modelling of the primary response.

To characterize the V̇O_2_ response during the 1 MT, data were reduced to a 5-s stationary average. Moreover, for better comparing the groups we used VO_2_ relative to VO_2_max, and not the absolute VO_2_, because during supramaximal exercise the VO_2_ response drives VO_2_ to reach their maximum value (i.e. VO_2_max) [22]. Thus, comparing supramaximal VO_2_ response in subjects with different VO_2_ amplitude could be misleading with respect to interpreting possible training-induced on VO_2_ responses. Therefore, the comparisons using VO_2_ relative to VO_2_max are more appropriate to analyze the VO_2_ response as it provides an indication of how fast is the changes in VO_2_ towards their predict asymptotic amplitude, that for supramaximal exercise is VO_2_max. In addition, the peak of V̇O_2_ (V̇O_2_peak) and the time to achieve the V̇O_2_peak (tV̇O_2_peak) were calculated based on the highest V̇O_2_ value. The possible V̇O_2_ decrease was determined from the difference between the V̇O_2_ value at 30 s and the end-exercise V̇O_2_. Finally, the total O_2_ consumed was determined as the time integral above the V̇O_2_rest for the 5-s V̇O_2_ values. The O_2_ consumed during the first and second half of the 1MT was also calculated.

### Ventilatory variables

During the 1MT and 110%MAV, the peak value of minute ventilation, tidal volume, and breathing frequency (i.e., the V̇Epeak, VTpeak, and BFpeak, respectively) were calculated based on highest 5-s average value during all the tests. Moreover, the possible declines in these parameters during the 1MT were determined from the difference between the value at 30 s and the end-exercise value.

### Statistical analysis

The results are expressed as mean ± SD. The Gaussian distribution of data was verified by the Kolmogorov–Smirnov test (with Lilliefors’ correction). In all cases, data were found to be acceptably normal. An unpaired t-test was used to assess the differences between groups for the parameters estimated from the V̇O_2_ responses and the metabolic parameters. A two-way analysis of variance (ANOVA) (group − time) with repeated measures on the time was used to assess the differences in the V̇O_2_ over time during 1 MT. When significant differences were observed, post hoc analyses were performed using Bonferroni corrections. The relationships between the V̇O_2_ parameters and the other variables obtained during the study were analysed merging all subjects into a single group and by using Pearson’s correlation coefficient (r). Statistical significance was set at p < 0.05.

## Results

### Incremental test and 1-Min all-out performance

Table 1 presents mean ± SD of the incremental test parameters and performance parameters during the 1MT. Compared to the SPRs, the ENDs had a higher MAV (*p* < 0.001) and V̇O_2_max (*p* < 0.001), which corresponded to a difference of 17 and 14%, respectively. On the other hand, the SPRs had a faster V_max_ (*p* < 0.001) and V_mean_ (*p* < 0.001), which corresponded to a difference of 16 and 8% compared to the ENDs, respectively.

**Table 1:** The mean ± SD of the incremental test data and performance parameters during 1 MT for sprinters (SPRs) and endurance runners (ENDs).

	ENDs	SPRs
V̇O_2_max (ml.kg^-1^.min^-1^)	59.6 ± 2.6*	51.5 ± 2.9
MAV (m.s^-1^)	5.1 ± 0.4*	4.6 ± 0.2
V_max_(m.s^-1^)	7.8 ± 0.7*	9.2 ± 0.6
V_mean_(m.s^-1^)	6.6 ± 0.3*	7.1 ± 0.4

MAV, maximal aerobic velocity; V̇O_2_max, maximum oxygen uptake; V_max_ and V_mean_, maximum and mean velocity during 1-min all-out.

* significant difference between groups (*p* < 0.001).

### V̇O_2_ analysis and metabolic data

**Fig 1:**
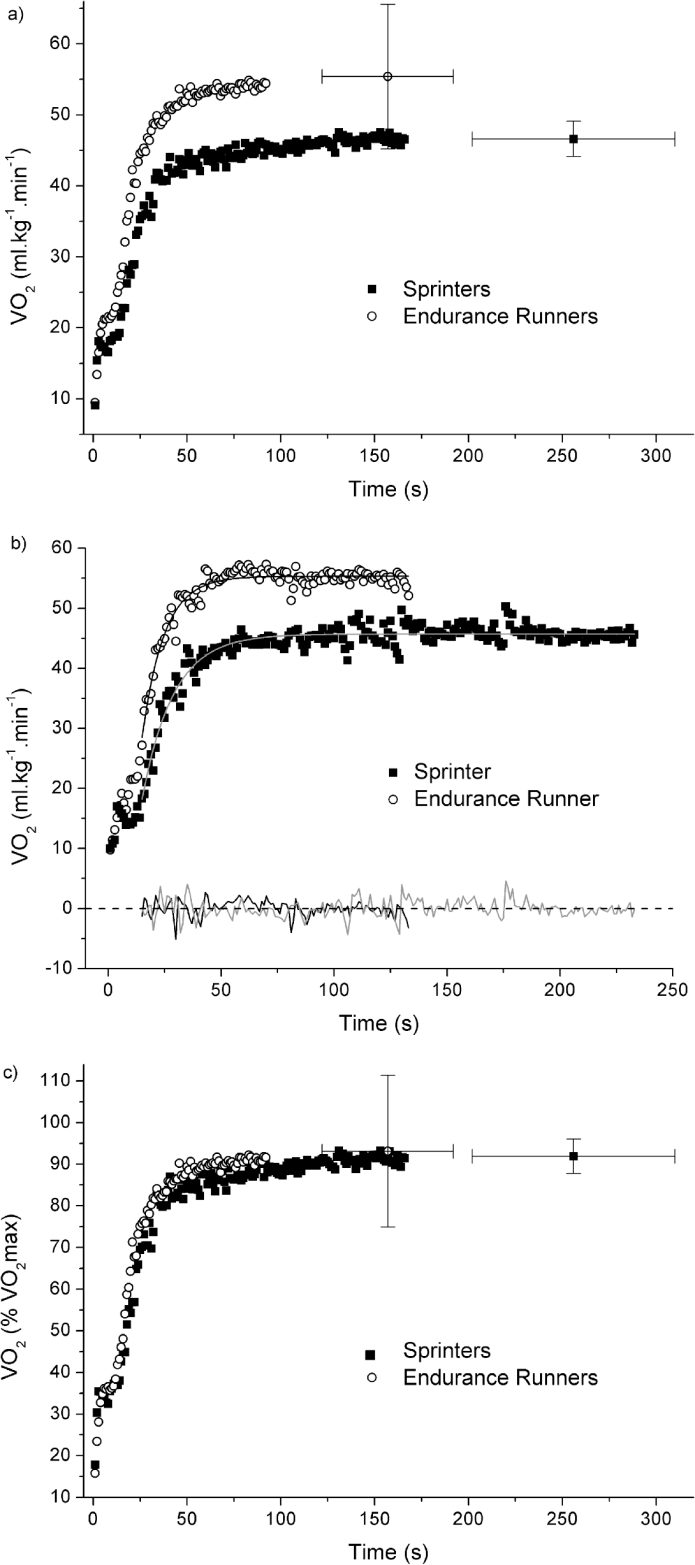
Pulmonary V̇O_2_ response during the 110%MAV test for group mean data (a,b) and for a representative subject in each group (c). VO_2_ was expressed in absolute and relative terms (%VO_2_max) in Fig 1A and 1B, respectively. In Fig 1A and 1B, data were matched at the shortest time to exhaustion recorded in each group. Moreover, the mean ± SD of the asymptote (i.e. amplitude + V̇O_2_ rest) and time to exhaustion are also shown. In Fig 1C, the exponential fits of the data and the residuals were also illustrated.

The main results of the V̇O_2_ kinetics and blood parameters during the 110%MAV test are presented in Table 2. The V̇O_2_ kinetics during the 110%MAV was faster in the ENDs compared to SPRs (*p* = 0.009). Furthermore, the amplitude of the primary component was higher in the ENDs compared to SPRs (*p* = 0.042). Fig 1 shows the pulmonary V̇O_2_ response during the 110%MAV test.

**Table 2:** The V̇O_2_ kinetic parameters and blood parameters during the 110%MAV test for sprinters (SPRs) and endurance runners (ENDs).

	ENDs	SPRs	*P* value
V̇O_2_ rest (ml.kg^-1^.min^-1^)	6.3 ± 0.8	6.7 ± 1.1	.42
A (ml.kg^-1^.min^-1^)	49.0 ± 10.0*	40.2 ± 2.4	.042
TD (s)	6.8 ± 3.4	4.5 ± 5.1	.28
τ (s)	12.7 ± 3.0*	19.3 ± 5.6	.009
∆BLC (mmol.l^-1^)	10.3 ± 3.5	10.4 ± 2.8	.92
∆pH	-0.26 ± 0.07	-0.24 ± 0.09	.81

A, TD and τ are the amplitude, time delay and time constant estimated from V̇O2 kinetics. ∆BLC and ∆pH is the difference between pre test and peak exercise values of blood lactate concentration and pH, respectively.

*Significant difference between groups (*p* < 0.05).

The main results of the V̇O_2_ responses and blood parameters during the 1 MT are presented in Table 3. Compared to the ENDs, the SPRs had a significantly lower V̇O_2_peak, tV̇O_2_peak and O_2_ consumed during 1 MT (*p* < 0.05). In addition, when the O_2_ consumed was divided into two parts, there was significant difference between the groups in the second half (44.5 + 5.7 and 32.5 + 4.3 ml.Kg^-1^ for ENDs and SPR, respectively; *p* < 0.001), but not in the first half of the 1 MT (30.8 + 3.6 and 28.0 + 2.4 ml.Kg^-1^ for ENDs and SPR, respectively; *p* = 0.089). There was a V̇O_2_ decrease for all SPRs and for only one endurance runner during 1 MT, which resulted in a significantly higher V̇O_2_ decrease in SPRs (*p* < 0.001).

**Table 3:** The V̇O_2_ responses and blood parameters during the 1-min all-out test for sprinters (SPRs) and endurance runners (ENDs).

	ENDs	SPRs	*P* Value
V̇O_2_ rest (ml.kg^-1^.min^-1^)	6.4 ± 0.5	6.5 ± 0.7	.92
V̇O_2_peak (ml.kg^-1^.min^-1^)	52.6 ± 6.0*	42.4 ± 4.4	.002
V̇O_2_peak (%V̇O_2_max)	88.2 ± 8.4	82.8 ± 5.9	.157
Total O_2_ consumed (ml.kg^-1^)	37.6 ± 4.0*	30.3 ± 3.3	.001
tV̇O_2_peak (s)	40.6 ± 6.8*	28.8 ± 6.4	.002
V̇O_2_ decrease (ml.kg^-1^.min^-1^)	-1.8 ± 2.3*	3.5 ± 2.3	< .000
∆BLC (mmol.l^-1^)	12.4 ± 2.9*	19.2 ± 2.2	< .001
∆pH	-0.29 ± 0.07*	-0.43 ± 0.06	.002

V̇O_2_peak was determined as the 15-s rolling average; tV̇O_2_peak is the time to achieve V̇O_2_peak; V̇O_2_ decrease is the difference between the V̇O_2_ value at 30 s and the end-exercise V̇O_2_; O_2_ consumed was determined as the time integral above the V̇O_2_rest for the 5-s V̇O_2_ values; ∆BLC and ∆pH is the difference between pre test and peak exercise values of blood lactate concentration and pH, respectively.

*Significant difference between groups (*p* < 0.05)

**Fig 2:**
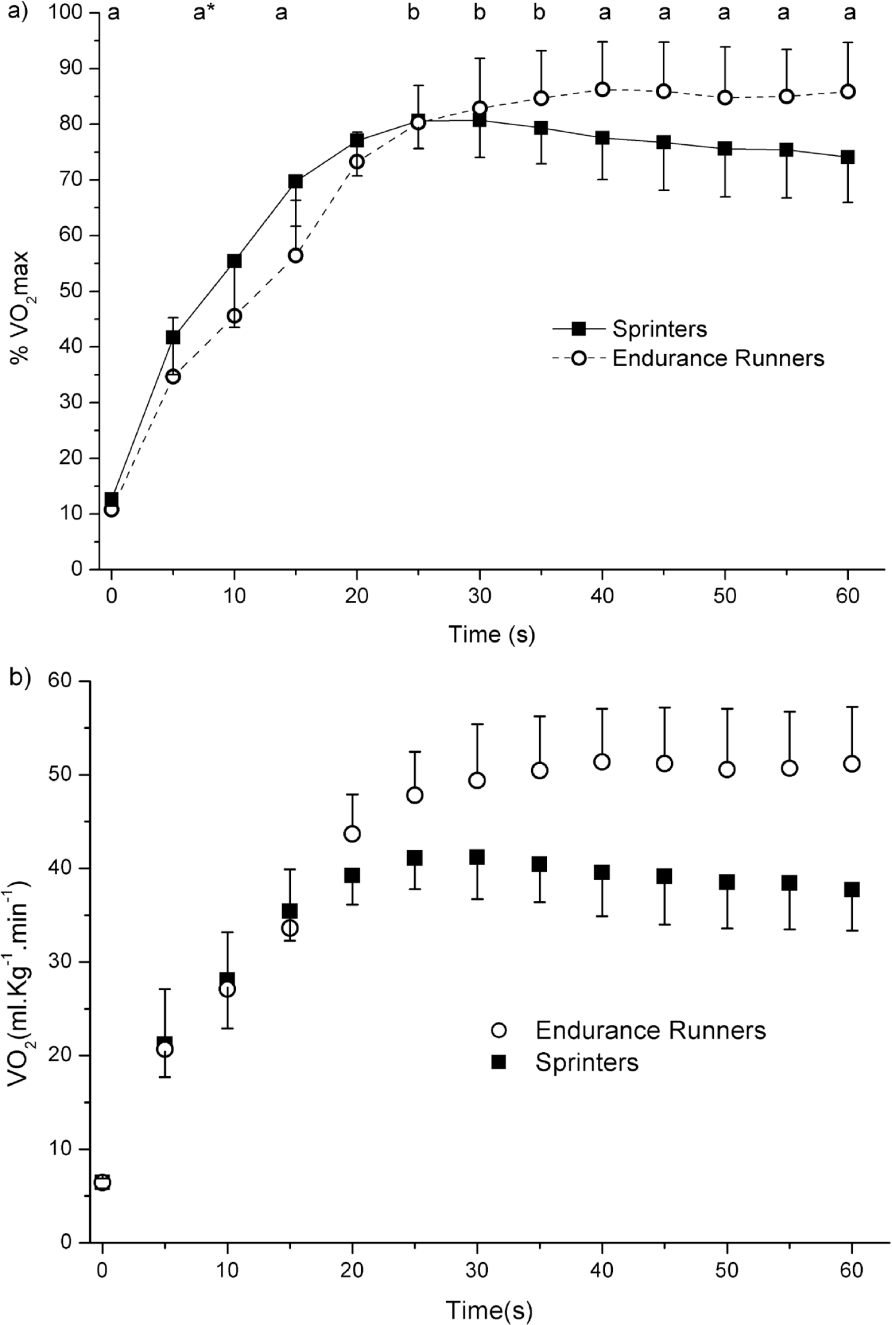
Time course of the V̇O_2_ during the 1 min all-out running test in sprinters and endurance runners. VO_2_ was expressed in relative (%VO_2_max) and absolute terms in Fig 1A and 1B, respectively. Statistical analysis was only performed on relative terms. ^a^significant difference between groups (*p* < 0.05); ^a*^statistical trend for a higher V̇O_2_ in sprinters (*p* = 0.09); ^b^V̇O_2_ significantly higher than end-exercise V̇O_2_ in sprinters (*p* < 0.05).

Fig 2 shows the V̇O_2_ response during 1 MT. The ANOVA revealed a significant time and interaction effect (*p* ≤ 0.01). The V̇O_2_ relative to the V̇O_2_max was significantly higher in the first V̇O_2_ points (first and fourth time point, *p* < 0.05) and was significantly lower in the latest V̇O_2_ points (from the ninth until the last time point, *p* < 0.05) in SPRs compared to the ENDs. Furthermore, there was a statistical trend for a higher V̇O_2_ in the third time point in SPRs (*p* = 0.09). Finally, the sprinters was the only group in which the last V̇O_2_ time point was significantly lower than other V̇O_2_ time point (i.e., sixth, seventh and eighth time point, *p* < 0.05).

The SPRs had a higher ∆BLC and ∆pH during the 1MT (*p* < 0.01) compared to the ENDs. However, during the 110%MAV, no significant differences in the ∆BLC and ∆pH were observed.

### Ventilatory parameters

The 1MT peak values of V̇E, VT, and BF were 145 ± 10 and 139 ± 18 l.min^-1^, 2.39 ± 0.32 and 2.38 ± 0.56 l.min^-1^ and 76 ± 15 and 75 ± 13 breaths.min^-1^ for the ENDs and SPRs, respectively, with no significant differences observed between groups (*p* > 0.1). During the 1MT, a decrease in V̇E was observed only in four SPRs and two ENDs. Furthermore, eight SPRs and seven ENDs had a decrease in BF, but only two SPRs had a decrease in VT.

### Correlations

The time constant of the exponential response during the 110%MAV was significantly correlated with the V̇O_2_max (r = -0.58) and MAV (r = -0.65). The tV̇O_2_peak during the 1MT was correlated with the ∆BLC (r = -0.74), ∆pH (r = 0.80), V_max_ (r = -0.61) and V_mean_ (r = -0.56). Finally, the V̇O_2_ decrease and percentage of the V̇O_2_max achieved during the 1MT was correlated with the ∆pH (r = -0.66 and 0.82, respectively), ∆BLC (r = 0.74 and -0.71, respectively), V_max_ (r = 0.69 and -0.63) and V_mean_ (r = 0.56 and -0.59).

## Discussion

The purpose of the present study was to compare the V̇O_2_ responses during all-out and supramaximal constant-velocity running exercises in subjects with different training background. The important finding of this investigation was that, while the endurance athletes had a faster V̇O_2_ response during supramaximal constant-velocity running exercise, the sprinters had a faster V̇O_2_ response during all-out exercise. In addition, the sprinters presented a decrease in V̇O_2_ in the last part of the 1 MT.

Previous studies and the current one using constant-intensity exercise have reported faster V̇O_2_ responses in endurance athletes compared to nonathletic or other athletic populations and after endurance training [4, 5, 23]. Accordingly, some studies showed experimentally that the time constant of V̇O_2_ kinetics decreases with the increase of the mitochondrial content [24, 25].

During the 1MT, modeling the overall V̇O_2_ kinetics was not considered suitable, particularly because of the V̇O_2_ decrease and short time window. Therefore, it was analyzed only tV̇O_2_peak and V̇O_2_ at each 5 s average time point. In contrast to constant running exercise, the comparison of such measures between the groups showed a faster V̇O_2_ response in SPRs. Indeed, for supramaximal exercises V̇O_2_ kinetics drives V̇O_2_ toward the overall energy requirement, but it is never reached since the increase in V̇O_2_ is constrained by V̇O_2_max. Therefore, for supramaximal intensities, the higher is the exercise intensity, the shorter is the time to achieve V̇O_2_max when the exercise duration permits [26]. In addition, the postulated model of mitochondrial respiratory control suggests that the rate of adjustment of oxidative phosphorylation during the on-transient kinetics would be mainly linked by the rate of muscle ATP hydrolysis. Therefore, the level of cellular metabolic controllers is intimately linked to mitochondrial respiration through feedback control [13, 27, 28]. In this context, some studies have shown that the rate of phosphocreatine (PCr) breakdown in the active muscles is proportional to the changes per unit time in V̇O_2_ [13, 14]. This suggest that, in the present study, the higher speed of SPRs during all-out exercise could induce higher PCr breakdown and, consequently, have led to a faster V̇O_2_ response during all-out exercise. Therefore, considering the similar difference between groups in aerobic and anaerobic parameters, these putative factors show that despite the fact that capillary density and mitochondrial content affect the V̇O_2_ kinetics, it seems to be more dependent, at least for supramaximal intensities, on the change in the intermediate metabolite concentration per unit change in time [28].In addition to these mechanisms, the correlation of the ∆BLC and ∆pH with tV̇O_2_peak during the 1MT were consistent with a notion that lactic acidosis may increase muscle perfusion and O_2_ delivery and thus speed-up the V̇O_2_ response [29, 30], though there are some evidences showing that the O_2_ delivery to muscle does not seem to be a limiting factor for V̇O_2_ response [13]

SPRs achieved the V̇O_2_peak faster during all-out running exercise, yet they obtained a lower O_2_ consumed, representing <24% that consumed by the ENDs. Thus, the higher O_2_ consumed during all-out exercise in ENDs seems to be linked to higher V̇O_2_ amplitude along the transition from rest to exercise as well as a meaningful V̇O_2_ decrease for SPRs during the latter half of the test. These results highlight that, although anaerobic training is highly important to sprint performance, the aerobic adaptation can assist for improving the long sprint performance. The lower difference between SPRs and ENDs for V_mean_ compared with V_max_ (from 16 to 8%) also corroborates with this premise. In this context, some studies have showed that a greater aerobic contribution has impacted positively during sprint tests [12, 31], as well as, sprint running performance seem to be determined by anaerobic parameters, in conjunction with V̇O_2_max [32],

The V̇O_2_ decrease has previously been reported during long sprint running events [2, 8, 9]. In the present study, all SPRs had a negative value for the difference between the V̇O_2_ value at 30 s and the end-exercise V̇O_2,_ indicating a V̇O_2_ decrease during the second half of the test. In contrast, the mean value of this difference was positive in ENDs. Some studies have indicated that a large glycolytic contribution may partially explain the V̇O_2_ decrease during short and intense running exercise [9, 18], and under *in vitro* conditions acidosis has been shown to inhibit oxidative phosphorylation in contracting muscles [33]. Therefore, our findings were expected since SPRs had higher metabolites derived from glycolytic metabolism during all-out exercise. The significant correlations of the ∆pH and ∆BLC with the V̇O_2_ decrease during all-out running exercise help support this premise. Additionally, it was suggested that a reduction of inspiratory muscle strength, which would be evidenced by a decrease in VT, can result in an V̇O_2_ decrease during exhaustive exercise [34]; however in the present study, only two SPRs had VT decline despite the fact that all SPRs had a V̇O_2_ decrease.

Burnley and Jones [35] and Di Prampero [10] have highlighted the importance of the V̇O_2_ response for supramaximal performance. Based on this, several studies have showed that pacing strategy may have marked impact on V̇O_2_ responses and, consequently, effects on exercise performance [9, 12, 36]. These studies and the current one showed that fast and/or all-out pacing strategy is necessary to reach a high relative V̇O_2_ in a short time, and to provide the best performance [9, 12, 36]. The present study also showed that there is a V̇O_2_ decrease in the last part the all-out test only for athletes with elevated anaerobic ATP turnover (i.e. sprinters). Although the inhibition of the oxidative phosphorylation by low value of pH seems to be harmful, successful performance on the long sprint running requires full use of the buffering capacity [37], and therefore critical final values of pH [38]. Since we are not able to evaluate the effect these responses on competitive races, further studies should be conducted, perhaps investigating the interaction between different fast pacing strategies and training status in a “real-life” running performance.

In conclusion, our findings showed that even though endurance runners had a higher aerobic power and O_2_ consumed during all-out and supramaximal constant-velocity running exercise, the sprinters had a faster V̇O_2_ response when the maximum intensity was required. Therefore, the ability to speed-up the initial V̇O_2_ response during all-out supramaximal running exercise seems to be more dependent on the change of the intermediate metabolite concentration per unit change in time than the aerobic background. In addition, our findings also showed that a high maximum intensity during all-out running exercise seems to lead to a higher decrease in the V̇O_2_ during the last part of the exercise, likely due to a higher accumulation of anaerobic metabolic by-products.

## References

[1] Spencer MR, Gastin PB. Energy system contribution during 200- to 1500-m running in highly trained athletes. Med Sci Sports Exerc. 2001;33(1):157–62. Epub 2001/02/24. . 11194103 10.1097/00005768-200101000-00024

[2] Thomas C, Hanon C, Perrey S, Le Chevalier JM, Couturier A, Vandewalle H. Oxygen uptake response to an 800-m running race. Int J Sports Med. 2005;26(4):268–73. Epub 2005/03/30. 10.1055/s-2004-820998 . 15795810

[3] Murgatroyd SR, Ferguson C, Ward SA, Whipp BJ, Rossiter HB. Pulmonary O2 uptake kinetics as a determinant of high-intensity exercise tolerance in humans. J Appl Physiol. 2011;110(6):1598–606. Epub 2011/03/19. doi: japplphysiol.01092.2010 [pii] 10.1152/japplphysiol.01092.2010 . 21415174

[4] Cleuziou C, Perrey S, Lecoq AM, Candau R, Courteix D, Obert P. Oxygen uptake kinetics during moderate and heavy intensity exercise in humans: the influence of hypoxia and training status. Int J Sports Med. 2005;26(5):356–62. Epub 2005/05/17. 10.1055/s-2004-821158 . 15895318

[5] Berger NJ, Jones AM. Pulmonary O2 uptake on-kinetics in sprint- and endurance-trained athletes. Appl Physiol Nutr Metab. 2007;32(3):383–93. Epub 2007/05/19. doi: h06-109 [pii] 10.1139/H06-109 . 17510672

[6] Berger NJ, Rittweger J, Kwiet A, Michaelis I, Williams AG, Tolfrey K, et al Pulmonary O2 uptake on-kinetics in endurance- and sprint-trained master athletes. Int J Sports Med. 2006;27(12):1005–12. Epub 2006/04/14. 10.1055/s-2006-923860 . 16612739

[7] Sandals LE, Wood DM, Draper SB, James DV. Influence of pacing strategy on oxygen uptake during treadmill middle-distance running. Int J Sports Med. 2006;27(1):37–42. Epub 2006/01/03. 10.1055/s-2005-837468 . 16388440

[8] Hanon C, Lepretre PM, Bishop D, Thomas C. Oxygen uptake and blood metabolic responses to a 400-m run. Eur J Appl Physiol. 2010;109(2):233–40. Epub 2010/01/12. 10.1007/s00421-009-1339-4 . 20063105

[9] Hanon C, Thomas C. Effects of optimal pacing strategies for 400-, 800-, and 1500-m races on the VO2 response. J Sports Sci. 2011;29(9):905–12. Epub 2011/05/07. doi: 937309980 [pii] 10.1080/02640414.2011.562232 . 21547833

[10] di Prampero PE. Factors limiting maximal performance in humans. Eur J Appl Physiol. 2003;90(3–4):420–9. 10.1007/s00421-003-0926-z . 12910345

[11] Maronski R. Minimum-time running and swimming: an optimal control approach. J Biomech. 1996;29(2):245–9. Epub 1996/02/01. doi: 0021929095000410 [pii]. . 8849819 10.1016/0021-9290(95)00041-0

[12] Turnes T, Salvador AF, Lisboa FD, de Aguiar RA, Cruz RS, Caputo F. A fast-start pacing strategy speeds pulmonary oxygen uptake kinetics and improves supramaximal running performance. PLoS One. 2014;9(10):e111621 10.1371/journal.pone.0111621 25360744 PMC4216092

[13] Poole DC, Jones AM. Oxygen uptake kinetics. Compr Physiol. 2012;2(2):933–96. Epub 2012/04/01. 10.1002/cphy.c100072 . 23798293

[14] Moxnes JF, Hausken K, Sandbakk O. On the kinetics of anaerobic power. Theoretical biology & medical modelling. 2012;9:29 10.1186/1742-4682-9-29 22830586 PMC3548746

[15] Cleuziou C, Perrey S, Borrani F, Lecoq AM, Candau R, Courteix D, et al Dynamic responses of O2 uptake at the onset and end of exercise in trained subjects. Can J Appl Physiol. 2003;28(4):630–41. Epub 2003/09/10. . 12959096 10.1139/h03-048

[16] Johansen L, Quistorff B. 31P-MRS characterization of sprint and endurance trained athletes. Int J Sports Med. 2003;24(3):183–9. Epub 2003/05/13. 10.1055/s-2003-39085 . 12740736

[17] Ufland P, Ahmaidi S, Buchheit M. Repeated-sprint performance, locomotor profile and muscle oxygen uptake recovery: effect of training background. Int J Sports Med. 2013;34(10):924–30. Epub 2013/06/07. 10.1055/s-0033-1333719 . 23740343

[18] Billat V, Hamard L, Koralsztein JP, Morton RH. Differential modeling of anaerobic and aerobic metabolism in the 800-m and 1,500-m run. J Appl Physiol. 2009;107(2):478–87. Epub 2009/05/30. doi: 91296.2008 [pii] 10.1152/japplphysiol.91296.2008 . 19478190

[19] Granier P, Mercier B, Mercier J, Anselme F, Prefaut C. Aerobic and anaerobic contribution to Wingate test performance in sprint and middle-distance runners. Eur J Appl Physiol Occup Physiol. 1995;70(1):58–65. Epub 1995/01/01. . 7729439 10.1007/BF00601809

[20] Scott CB, Roby FB, Lohman TG, Bunt JC. The maximally accumulated oxygen deficit as an indicator of anaerobic capacity. Med Sci Sports Exerc. 1991;23(5):618–24. Epub 1991/05/01. . 2072841

[21] Kindermann W, Keul J, Huber G. Physical exercise after induced alkalosis (bicarbonate or tris-buffer). Eur J Appl Physiol Occup Physiol. 1977;37(3):197–204. Epub 1977/10/31. . 913385 10.1007/BF00421775

[22] di Prampero PE, Ferretti G. The energetics of anaerobic muscle metabolism: a reappraisal of older and recent concepts. Respir Physiol. 1999;118(2–3):103–15. Epub 2000/01/27. . 10647856 10.1016/s0034-5687(99)00083-3

[23] Caputo F, Denadai BS. Effects of aerobic endurance training status and specificity on oxygen uptake kinetics during maximal exercise. Eur J Appl Physiol. 2004;93(1–2):87–95. Epub 2004/07/13. 10.1007/s00421-004-1169-3 . 15248068

[24] Phillips SM, Green HJ, MacDonald MJ, Hughson RL. Progressive effect of endurance training on VO2 kinetics at the onset of submaximal exercise. J Appl Physiol. 1995;79(6):1914–20. Epub 1995/12/01. . 8847253 10.1152/jappl.1995.79.6.1914

[25] Glancy B, Barstow T, Willis WT. Linear relation between time constant of oxygen uptake kinetics, total creatine, and mitochondrial content in vitro. Am J Physiol Cell Physiol. 2008;294(1):C79–87. Epub 2007/10/19. doi: 00138.2007 [pii] 10.1152/ajpcell.00138.2007 . 17942641

[26] Hill DW, Stevens EC. VO2 response profiles in severe intensity exercise. J Sports Med Phys Fitness. 2005;45(3):239–47. Epub 2005/10/19. . 16230972

[27] Bose S, French S, Evans FJ, Joubert F, Balaban RS. Metabolic network control of oxidative phosphorylation: multiple roles of inorganic phosphate. J Biol Chem. 2003;278(40):39155–65. Epub 2003/07/23. doi: 10.1074/jbc.M306409200 M306409200 [pii]. . 12871940 10.1074/jbc.M306409200

[28] Korzeniewski B, Zoladz JA. Factors determining the oxygen consumption rate (VO2) on-kinetics in skeletal muscles. Biochem J. 2004;379(Pt 3):703–10. Epub 2004/01/28. doi: 10.1042/BJ20031740 BJ20031740 [pii]. 14744260 10.1042/BJ20031740 PMC1224118

[29] Poole DC, Barstow TJ, McDonough P, Jones AM. Control of oxygen uptake during exercise. Med Sci Sports Exerc. 2008;40(3):462–74. Epub 2008/04/02. 10.1249/MSS.0b013e31815ef29b . 18379208

[30] Grassi B. Regulation of oxygen consumption at exercise onset: is it really controversial? Exerc Sport Sci Rev. 2001;29(3):134–8. . 11474962 10.1097/00003677-200107000-00009

[31] Foster C, Snyder AC, Thompson NN, Green MA, Foley M, Schrager M. Effect of pacing strategy on cycle time trial performance. Med Sci Sports Exerc. 1993;25(3):383–8. Epub 1993/03/01. . 8455455

[32] Dal Pupo J, Arins FB, Antonacci Guglielmo LG, Rosendo da Silva RC, Moro AR, Dos Santos SG. Physiological and neuromuscular indices associated with sprint running performance. Res Sports Med. 2013;21(2):124–35. Epub 2013/04/02. 10.1080/15438627.2012.757225 . 23541099

[33] Jubrias SA, Crowther GJ, Shankland EG, Gronka RK, Conley KE. Acidosis inhibits oxidative phosphorylation in contracting human skeletal muscle in vivo. J Physiol. 2003;553(Pt 2):589–99. Epub 2003/09/30. 10.1113/jphysiol.2003.045872jphysiol.2003.045872 [pii]. 14514869 PMC2343560

[34] Gallagher CG, Hof VI, Younes M. Effect of inspiratory muscle fatigue on breathing pattern. J Appl Physiol (1985). 1985;59(4):1152–8. Epub 1985/10/01. . 4055595 10.1152/jappl.1985.59.4.1152

[35] Jones AM, Burnley M. Oxygen uptake kinetics: an underappreciated determinant of exercise performance. Int J Sports Physiol Perform. 2009;4(4):524–32. . 20029103 10.1123/ijspp.4.4.524

[36] Bishop D, Bonetti D, Dawson B. The influence of pacing strategy on VO2 and supramaximal kayak performance. Med Sci Sports Exerc. 2002;34(6):1041–7. Epub 2002/06/06. . 12048335 10.1097/00005768-200206000-00022

[37] Ward-Smith AJ. Aerobic and anaerobic energy conversion during high-intensity exercise. Med Sci Sports Exerc. 1999;31(12):1855–60. . 10613440 10.1097/00005768-199912000-00025

[38] Foster C, Schrager M, Snyder AC, Thompson NN. Pacing strategy and athletic performance. Sports Med. 1994;17(2):77–85. . 8171225 10.2165/00007256-199417020-00001

